# Explanatory preferences for complexity matching

**DOI:** 10.1371/journal.pone.0230929

**Published:** 2020-04-21

**Authors:** Jonathan B. Lim, Daniel M. Oppenheimer

**Affiliations:** 1 School of Business, UC Riverside, Riverside, California, United States of America; 2 Social and Decision Sciences, Carnegie Mellon University, Pittsburgh, Pennsylvania, United States of America; University of Klagenfurt, AUSTRIA

## Abstract

People are adept at generating and evaluating explanations for events around them. But what makes for a satisfying explanation? While some scholars argue that individuals find simple explanations to be more satisfying (Lombrozo, 2007), others argue that complex explanations are preferred (Zemla, et al. 2017). Uniting these perspectives, we posit that people believe a satisfying explanation should be as complex as the event being explained–what we term the complexity matching hypothesis. Thus, individuals will prefer simple explanations for simple events, and complex explanations for complex events. Four studies provide robust evidence for the complexity-matching hypothesis. In studies 1–3, participants read scenarios and then predicted the complexity of a satisfying explanation (Study 1), generated an explanation themselves (Study 2), and evaluated explanations (Study 3). Lastly, in Study 4, we explored a different manipulation of complexity to demonstrate robustness across paradigms. We end with a discussion of mechanisms that might underlie this preference-matching phenomenon.

## 1. Introduction

People regularly seek explanations for their experiences. A family may wonder why they didn’t enjoy their dinner at the local restaurant, a moviegoer may ask why she didn’t like the new hit film, and a student may consider why his expensive SAT prep class didn’t raise his SAT score. While there are dozens, even hundreds, of possible explanations for these events, they will not all be equally satisfying. As a result, individuals must sift through all of these potential explanations to arrive at a suitable answer: the waiter was extremely rude, the movie’s plot was boring, and the prep class went over basic techniques the student already knew. However, this raises the question: how were these conclusions reached, and why were they judged as being more acceptable than other possible explanations? More broadly, the question becomes: what makes an explanation satisfying?

A satisfying explanation encompasses more than just accuracy or believability (although both are necessary). For example, when thinking about why the San Francisco Forty-Niners lost the 2020 Super Bowl, most people would agree that the explanation “because they scored fewer points” is accurate, but it still remains unsatisfyingly tautological. Similarly, a play-by-play recounting of the game would be believable, but would still be unsatisfying because it does not seem to account for the larger reasons why the Rams may have lost. Thus, while much work has explored when people find explanations believable [1–2], that is not sufficient for understanding what makes an explanation compelling.

In this work, we propose that one important cue that individuals use in identifying a satisfying explanation is its complexity. Specifically, the current work advances the complexity-matching hypothesis: people will find an explanation more satisfying when it matches the complexity of its precipitating event. Individuals will tend to prefer a simple explanation for a simple event, and a complex explanation for a complex event.

## 2. Theoretical development

### Explanatory virtues

Within the domains of philosophy and psychology, much work has explored the characteristics of a satisfying explanation. Philosophers were among the first to ponder this issue [3–4], coining the term “explanatory virtues” to classify those traits inherent in a satisfying explanation. While a variety of different qualities have been considered, simplicity has traditionally been the virtue given the most attention by scholars [5–10]. Aristotle proposed that “we may assume the superiority *ceteris paribus* of the demonstration which results from fewer postulates or hypotheses” [11], while William of Occam famously postulated that “entities are not to be multiplied beyond necessity,” forming the basis for his famous razor [12]. More recently, Albert Einstein once surmised that “if you can’t explain it simply, you don’t understand it well enough,” and the *FBI Law Enforcement Bulletin*, in providing guidance on judging criminal acts, advised that “the least complicated explanation of an event is usually the correct one” [13].

Though simplicity has been much discussed, it is (ironically) a complex, multifaceted construct that has eluded a standard definition by scholars. However, there are two characteristics that are central to most conceptualizations of simplicity: parsimony (the number of elements described) and uniformity (the consistency of relationships among these elements) [11–12]. These factors can characterize simplicity of both events [5] and their explanations [14]. Simpler events would thus feature fewer causally-relevant details, and greater consistency among these details. Simpler explanations would contain fewer explanatory components and more consistent relationships among them.

Despite disagreement on the formal definition of simplicity, there has nonetheless traditionally been widespread empirical agreement that simpler explanations are more satisfying than complex explanations [5–8, 10, 15–20]. Lombrozo (2007) [5] demonstrated this preference for simplicity using a paradigm representing much of the work on this topic. Participants were given several pieces of data to evaluate (e.g., an alien has sore minttels and purple spots) and were provided information regarding these data (Tritchet’s syndrome causes sore minttels and purple spots; Morad’s disease causes sore minttels; Humel infection causes purple spots). They were then asked to consider different explanations accounting for the data: a simple explanation that was more parsimonious (e.g., the alien has Tritchet’s syndrome) and a complex explanation that was less parsimonious (e.g., the alien has Morad’s disease and a Humel infection). Overall, participants consistently favored simpler explanations, and this finding held true even when the complex explanations featured a higher probability of occurrence. In fact, Lombrozo found that participants only changed their preferences when the complex explanations were at least ten times more probable than the simpler alternatives.

Furthermore, this preference for simplicity may actually have adaptive value, helping individuals to optimally evaluate explanations. For example, Forster and Sober (1994) [21] determined that for curve-fitting, simpler curves have less of a tendency to over-fit the data (i.e., to track both underlying stable patterns *and* noise) than do more complex curves. Additionally, in the realm of education, Kelly (2004) [20] argued that simplicity was a critical attribute in hypothesis generation, as simpler hypotheses often need to be modified less by users in order to fit various situations. Thus, simplicity can often guide students’ learning of new material, aiding them in formulating theories that are useful in a wide variety of contexts. As a result, it is little wonder that individuals adopt a preference for simplicity from a young age [16, 18].

### Boundary conditions on explanatory virtues

Despite the robust body of research demonstrating a preference for simplicity, recent work has begun to question whether simpler explanations are always better fits for the types of events that people experience in the real world [14]. Zemla and colleagues [14] have argued that when participants are given a contrived scenario, such as the alien example from Lombrozo (2007) [5], a simple explanation may indeed be most satisfying. However, this penchant for simplicity may actually be an artifact of the experimental paradigm used by previous researchers, as such artificial scenarios often do not approximate the types of situations individuals come across in real life, or recruit from semantic memory.

To investigate this possibility, Zemla and colleagues (2017) [14] gave participants a set of real-world questions (e.g., “Why isn’t China’s population decreasing if they’ve had a one-child policy for 35 years?”), along with explanations that were either simple, featuring few causes (more parsimonious):

A: “Ethnic minorities and rural populations are exempt from the rule” or

B: “Chinese are living longer on average, and wealthier couples can pay the fine associated with rule violation”

or complex, featuring more causes (less parsimonious):

AB: “Ethnic minorities and rural populations are exempt from the rule. Also, Chinese are living longer on average, and wealthier couples can pay the fine associated with rule violation”

In contrast with previous research, Zemla et al. (2017) [14] found that participants preferred complex explanations featuring more reasons and detail.

Indeed, while previous work has pointed to simplicity, breadth, and coherence as being qualities people look for in satisfying explanations, there has been growing evidence to demonstrate that this may not always be the case [22–27]. Instead, it appears that at times, people may be seeking out the exact *opposite* qualities in explanations. In addition to the aforementioned work by Zemla and colleagues (2017) [14], research by O’Keefe (1997; 1998; 1999) [22–24] has advanced the notion that a unanimous preference for simplicity may not even exist. Through a series of meta-analyses, O’Keefe (1999) [24] examined individuals’ views on one-sided versus two-sided arguments. While presenting both sides of an argument is clearly less simple than presenting a single perspective, O’Keefe (1999) [24] found a preference for these complex appeals. Going even further, when participants were given different types of two-sided arguments, they favored more complex arguments in which opposing views were actively refuted, as opposed to arguments in which such views were merely acknowledged. Additionally, participants exhibited a strong preference for well-developed arguments–those in which quantitative support was provided, the sources were identified and cited, and ideas were fully fleshed out and viewpoints made explicit–even if such arguments were longer and more complex (O’Keefe, 1997, 1998) [22–23]. Such findings from O’Keefe corroborate research showing that in certain situations, people prefer explanations that are longer [28–29] and that use more complex language [30].

Even young children may at times demonstrate a preference for complexity [31–33]. Bartsch and Wellman (1989) [31] asked kids to think about the reasons for the actions of hypothetical individuals (e.g., “Jane is looking for her kitten under the piano. Why is Jane doing that?”). Participants overwhelmingly favored psychological reasons (“She is trying to find her kitten”) over behavioral (“Jane always looks there”) or physical ones (“The wind blew her there”). Previous research [34] has shown that explanations invoking social systems tend to be more complex than those regarding physical systems; thus, the children in Bartsch and Wellman’s studies appealed to more complex explanations in order to account for the hypothetical actions. Interestingly, this finding extends beyond these simple actions into children’s preferred explanations of others’ emotions [35] and past experiences, as well [36].

### Explanatory virtues… or vices?

It thus appears that what should be considered an explanatory virtue may not be quite as simple (no pun intended) as previously theorized–people may not carry around a set of defined explanatory preferences that they inflexibly look for when evaluating explanations. Instead, they may base their explanatory preferences on the particulars of the situation at hand, creating a set of explanatory criteria that is suited to these contextual nuances. From this perspective, if an explanatory virtue is by definition the qualities individuals look for in a good explanation, then it stands to reason that what we classify as being a virtue may depend on the circumstances at hand, thus shifting on a case-by-case basis.

### Complexity-matching

One key situational variable that individuals may take into account is the complexity of the event to be explained. A large body of work by Lombrozo (2007) [5] and others has demonstrated that people prefer simplicity in explanation, while emerging research, as exemplified by Zemla and colleagues (2017) [14], is beginning to show that individuals may instead favor complexity. It could be that both sides are partially correct, and what people are really responding to is complexity in the environment. Notably, the events to be explained in Zemla et al. (2017) [14] were in and of themselves more complex than the simpler lab stimuli used by Lombrozo (2007) [5]. Thus, it could be the case that people’s preference for simplicity or complexity in explanations is actually moderated by the simplicity or complexity of the event needing to be explained. We term this the complexity-matching hypothesis: people prefer for an explanation to match its precipitating event in complexity.

The concept of matching has precedent in the causal reasoning literature. Research has shown that individuals prefer causes and effects to match in terms of magnitude [37–38] and physical appearance [39], with this preference for matching holding true even when the cause has no diagnostic value in predicting the effect [40]. Additional work has also shown that individuals, even children as young as six years old, tend to match a machine’s functional diversity to its inner complexity, with participants attributing a greater diversity of function as having been “caused” by greater complexity of parts [41].

To examine whether complexity matching could explain the apparent discrepancies in the literature, we re-analyzed data from the second study in Zemla et al. (2017) [14]. Zemla and colleagues had participants think about a series of real-world questions and rate how complex potential answers to these questions would need to be in order to be satisfying. Taking this one step further, in our re-analysis, we had a new group of participants rate the complexity of these original stimuli, to see if there were natural variations in complexity among these questions. We found that there were indeed variations, and in line with complexity matching, more complex questions (as rated by our participants) were deemed to require more complex answers (as rated by Zemla et al.’s participants). While the Zemla et al. stimuli were not designed to test complexity matching, and thus are not a fair experimental test of our hypothesis, it is nonetheless a promising indication that people may prefer matching complexity for events and explanations. We explore this phenomenon more rigorously in the following set of studies.

## 3. Overview of studies

The following package of studies examines the descriptive validity of the complexity-matching hypothesis. In Studies 1–3, we examined the parsimony criterion of complexity by systematically manipulating the number of details in an event that needed to be explained. Participants assessed the satisfactoriness of an explanation in three different ways: predicting the complexity of a satisfying explanation (Study 1), generating a satisfying explanation themselves (Study 2), and evaluating how satisfying a potential explanation was (Study 3). In Study 4, we sought converging evidence through use of the uniformity criterion of complexity, as we systematically manipulated the valence of scenario details to either be internally consistent or inconsistent. Across all studies, we find evidence for the complexity-matching hypothesis.

## 4. Study #1

### 4.1. Method

#### 4.1.1. Participants

Participants (n = 286) were recruited through Amazon’s Mechanical Turk platform. While some researchers have expressed concern over the data quality from Mechanical Turk samples [42], research on Mturk has shown to achieve psychometric standards [43], replicate classic results across an array of behavioral disciplines such as sociology [44], political science [45], and cognitive science [46]. In order to be conservative, sample size was based on a power level of 0.8 and an estimated eta-squared effect size of 0.02. The sample was 39% male, 61% female, with an average age of 37 years. There were no participant exclusions based on attention or data quality checks in this, or any other, experiments reported in this manuscript. However, participants were excluded from participating in more than one study.

Approval for this study, along with the rest of the studies in this paper, was approved by UCLA Institutional Review Board IRB#14–001791.

#### 4.1.2. Materials

A set of four scenarios, spanning both positive and negative events, was created: a company experiencing success, a university having a great academic year, a baseball team going through several rough seasons, and an employee failing at his job. In line with Lombrozo (2007) [5], we operationalized scenario complexity in terms of parsimony. Thus, for each scenario, we varied the number of relevant details that participants saw. In the complex version of each scenario, participants viewed three details describing the event, whereas in the simple version, participants saw a more parsimonious account featuring only one detail (See S1 Appendix). For example, in the university scenario, the complex version read:

“Friedman University has been having a great year. It was recently christened a top-twenty university by Canadian News & World Report, the first time the school had ever received such an honor. Additionally, upon graduation, 90% of Friedman’s senior class this year will either be employed or attending graduate school. On top of this, the entering freshman class looks to be very strong, with an average high school GPA of 3.98 (out of 4.00).”

The simple version of the scenario only contained one of the three details from the complex version. Three simple scenario versions were thus created, one for each of the three details described in the complex version. For example:

“Friedman University has been having a great year. It was recently christened a top-twenty university by Canadian News & World Report, the first time the school had ever received such an honor.”

The other three scenarios (company, baseball team, and employee) were similarly constructed, with one complex version and three simple versions of each scenario. Participants saw all four scenarios, but were randomly assigned to see only one of the four versions of each scenario.

For each scenario, participants answered the following question: “How complex do you think a satisfying explanation for this event will be?” [14]. Explanation complexity was assessed on a 1–9 Likert scale, anchored by 1 = Extremely Simple, 9 = Extremely Complex. As a manipulation check, participants were also asked to evaluate the complexity of the scenario that they had read, assessed on the same 1–9 Likert scale.

### 4.2. Results

All of our analysis code for this study, and all other studies in this manuscript have been posted online at: https://osf.io/f3pm4/. The dependent measures were analyzed through linear mixed effects regression, with scenario complexity (simple, complex) entered as a fixed factor. We also included scenario (university, company, employee, baseball team) as an additional fixed effect, so that we could test the interaction between scenario complexity and scenario. Participants were modeled as a random factor to control for repeated measurements, and scenario complexity was also added as a random factor to control for random assignment of participants.

The manipulation check showed that our manipulation of complexity was successful—participants viewed the complex scenarios (*M* = 5.67, *SD* = .97) as being more complex than the simple scenarios (*M* = 4.88, *SD* = 2.21), *χ*^*2*^(1) = 38.02, *p* < .001.

As predicted by the complexity matching account, analysis revealed a main effect of scenario complexity, *χ*^*2*^(1) = 28.04, *p* < .001. Participants rated the complex versions of the scenarios (*M* = 5.59, *SD* = 2.04) as requiring more complex explanations than the simple versions of the scenarios (*M* = 4.95, *SD* = 2.15). There was also a main effect of scenario, *χ*^*2*^(3) = 62.07, *p* < .001 (see Table 1). However, there was no interaction between scenario complexity and scenario, *χ*^*2*^(3) = 1.52, *p* = ns.

**Table 1:** Complexity of a satisfying explanation.

	Complex	Simple
MacGrady	5.35 (1.61)	4.63 (2.00)
Baseball	6.00 (1.89)	5.60 (1.94)
Employee	6.10 (2.10)	5.36 (2.19)
Friedman	4.93 (2.30)	4.21 (2.18)

Statistics represent mean(standard deviation).

As a side note, one could reasonably argue that the data should be classified as ordinal, as opposed to continuous, thus warranting a multilevel mixed-effects ordered logistic regression. Such analysis actually produces the same pattern of results: main effect of scenario complexity, *χ*^*2*^(1) = 27.94, *p* < .001, main effect of scenario, *χ*^*2*^(3) = 59.79, *p* < .001, and no interaction between scenario and scenario complexity, *χ*^*2*^(3) = 1.22, *p* = ns. However, because interpreting the log odds provided by multilevel mixed-effects ordered logistic regression is not as intuitive for the average reader, and because solid arguments could also be made for the data to be treated as continuous, we have decided to utilize linear mixed effects regression throughout this paper (unless otherwise noted).

### 4.3. Discussion

This study provided experimental evidence for the complexity-matching hypothesis, showing that people expect complex events to have more complex explanations than simple events. However, in real life, individuals rarely *predict* the complexity of a satisfying explanation (even though that is a common dependent measure in the literature). More typically, people are tasked with coming up with explanations, either for themselves or for others. In these cases, do individuals *generate* explanations matching the complexity of the precipitating event?

Previous literature has demonstrated that people often have difficulty accurately predicting their preferences [47–48]. Thus, it could be the case that even though people predict that they would prefer explanations matching the complexity of the precipitating events, their actual generated explanations may not follow suit. Study 2 thus examined whether people observe complexity matching when generating explanations for events.

## 5. Study #2

### 5.1. Method

#### 5.1.1. Participants

Participants (n = 201) were recruited from Mechanical Turk. The sample was 40% male, 60% female, with an average age of 36 years. Sample size was based on the same calculations as for Study 1. We also excluded participants who had completed the previous studies.

#### 5.1.2. Materials and procedure

The same materials from Study 1 were used. Once again, participants read through all four scenarios, but were randomly assigned to see only one version of each scenario. After viewing each scenario, participants were asked to “write a compelling explanation for the [scenario] that would make sense to you or to the average person reading about the [scenario].” As in Study 1, participants were randomly assigned to see either simple or complex versions of each scenario.

### 5.2. Results

#### 5.2.1. Coding

Two independent raters, who were blind to condition, read each explanation and assigned an intuitive complexity rating on a 1–10 scale (1 = Completely Simple, 10 = Completely Complex). The average of their ratings (*r* = .64) formed the “complexity rating” for each explanation. Two other independent raters, also blind to condition, counted the number of causes listed in each explanation, with the average of their scores (*r* = .88) defining the “number of causes” variable. Lastly, we acquired a word count and Flesch-Kincaid score for each explanation, both obtained from standard packages offered by Microsoft Word. The Flesch-Kincaid score is a measure of the level of education needed to understand any passage of text (e.g., a Flesch-Kincaid score of 6 means that an individual would need to have a sixth-grade level of education to comprehend the passage) [49]. If the complexity-matching hypothesis holds, then for complex scenarios, participants should write more complex explanations featuring i) a higher complexity rating, ii) more causes listed, iii) a higher word count, and iv) a higher Flesch-Kincaid score.

Linear mixed effects modeling was again used to analyze each of the four main dependent measures. While multivariate analysis was considered, the strong correlations between variables could create serious multicollinearity problems (See Table 2). As a result, we analyzed each measure independently. For each dependent measure, scenario (university, company, employee, baseball team) and scenario complexity (simple, complex) were both entered as fixed factors, as was the interaction between them. Once again, participants and scenario complexity were included as a random factors.

**Table 2:** Correlations between dependent measures.

	Complexity	Flesch-Kincaid	Number of causes	Word count
Complexity	1.00	0.36	0.45	0.87
Flesch-Kincaid	0.36	1.00	0.14	0.27
Number of causes	0.45	0.14	1.00	0.33
Word count	0.87	0.27	0.33	1.00

#### 5.2.2. Complexity rating

There was a significant main effect of scenario complexity, *χ*^*2*^(1) = 46.53, *p* < .001. Participants’ explanations were rated as being more complex in response to a complex scenario (*M* = 4.53, *SD* = 1.48), than a simple scenario (*M* = 4.04, *SD* = 1.23). There was no effect of scenario, *χ*^*2*^(3) = 4.10, *p* = ns (See Table 3), and no interaction between scenario and scenario complexity, *χ*^*2*^(3) = 3.81, *p* = ns.

**Table 3:** Complexity rating.

	Complex	Simple
MacGrady	3.94 (2.28)	3.63 (1.97)
Baseball	4.26 (2.20)	3.29 (1.39)
Employee	3.91 (1.67)	3.26 (1.47)
Friedman	3.80 (1.32)	3.43 (1.31)

Statistics represent mean(standard deviation).

#### 5.2.3. Flesch-Kincaid score

While Flesch-Kincaid scores fell in the predicted direction, the main effect for scenario complexity was not statistically significant, *χ*^*2*^(1) = 1.26, *p* = ns. The readability of participants’ explanations did not reliably change from complex scenarios (*M* = 8.57, *SD* = 3.08) to simple scenarios (*M* = 8.38, *SD* = 3.10), see S2 Appendix. There was a main effect of scenario, *χ*^*2*^(3) = 105.94, *p* < .001 (See Table 4), but no interaction between scenario and scenario complexity, *χ*^*2*^(3) = 3.25, *p* = ns.

**Table 4:** Flesch-Kincaid score.

	Complex	Simple
MacGrady	9.03 (2.68)	9.06 (2.34)
Baseball	8.60 (3.08)	8.59 (3.24)
Employee	7.24 (3.17)	6.38 (3.07)
Friedman	9.47 (2.97)	9.48 (2.76)

Statistics represent mean(standard deviation).

#### 5.2.4. Number of causes

Given that number of causes is classified as count data and would best be categorized under a Poisson distribution, a mixed effects Poisson regression model was used instead to analyze the dependent measure. Participants included slightly more causes when writing explanations for complex scenarios (*M* = 1.29, *SD* = .95) than for simple scenarios (*M* = 1.26, *SD* = .85), but this difference did not reach conventional standards for statistical significance, *χ*^*2*^(1) = 0.19, *p* = ns (see S2 Appendix). There was no main effect of scenario, *χ*^*2*^(3) = 6.01, *p* = ns (See Table 5), and the interaction between scenario and scenario complexity was once again not significant, *χ*^*2*^(3) = 0.39, *p* = ns.

**Table 5:** Number of causes.

	Complex	Simple
MacGrady	1.48 (1.26)	1.46 (1.07)
Baseball	1.21 (0.85)	1.20 (0.82)
Employee	1.29 (0.94)	1.17 (0.59)
Friedman	1.17 (0.62)	1.22 (0.84)

Statistics represent mean(standard deviation).

#### 5.2.5. Word count

Since word count also follows a Poisson distribution, a mixed effects Poisson regression model was once again used. There was a significant main effect of scenario complexity, *χ*^*2*^(1) = 6.11, *p* = .01. Participants wrote more words for complex scenarios (*M* = 30.90, *SD* = 20.58) than they did for simple scenarios (*M* = 26.37, *SD* = 16.70). There was also a main effect for scenario, *χ*^*2*^(3) = 30.83, *p* < .01 (See Table 6), but no interaction between scenario and scenario complexity, *χ*^*2*^(3) = 5.15, *p =* ns.

**Table 6:** Word count.

	Complex	Simple
MacGrady	31.36 (24.60)	27.89 (15.95)
Baseball	34.71 (22.28)	28.04 (16.80)
Employee	31.96 (20.47)	25.09 (18.32)
Friedman	25.80 (12.66)	24.40 (15.48)

Statistics represent mean(standard deviation).

### 5.3. Discussion

Study 2 provided converging evidence for the complexity-matching hypothesis, demonstrating that individuals generate explanations matching with their precipitating events in complexity. Specifically, complex events elicited explanations with significantly higher complexity ratings and word counts than did simple events. Additionally, while only two of the four dependent measures reached conventional levels of statistical significance, all four measures trended in the predicted direction. Most importantly, raters strongly and reliably perceived the participants’ explanations to be more complex for the complex scenarios and simpler for the simple scenarios, which suggests that complexity may come in forms that are hard to capture through simple numerical metrics such as Flesch-Kincaid scores.

The previous two studies have provided experimental evidence showing that people both predict and generate explanations matching in complexity with their precipitating events. However, generation remains an imperfect measure, as previous research has shown that individuals can have trouble generating explanations that others will find satisfying [50]. Thus, the next study relied on a third dependent measure: evaluation. Participants were shown explanations varying in complexity and asked to assess how satisfying they found the explanations.

## 6. Study #3

### 6.1. Method

#### 6.1.1. Participants

Participants (n = 523) were recruited from Mechanical Turk. The sample was 36% male and 64% female, with an average age of 36 years. As before, participants who had done either of the previous studies were not allowed to enroll in the current one.

#### 6.1.2. Materials and procedure

The same scenarios from Studies 1 and 2 were used. To reduce logistical complexity, we randomly selected two of the four possible scenarios (baseball team and university), and within each scenario, we used the complex version and one randomly selected simple version. This left us with four conditions.

To obtain explanations, we sampled from explanations generated by participants in Study 2. Doing so allows participants in the current study to evaluate explanations akin to what they would see in everyday life. As a result, it provides a sense of external validity complementing the other more rigorously-controlled studies. Further, this approach guards against experimenter bias in the creation of the stimuli [51].

As a result, within each condition (simple and complex), we examined the set of explanations that participants had generated, using the coders’ average complexity rating (which was assessed on a 1–10 scale), to identify the lowest-rated explanation (simplest) and the highest-rated explanation (most complex), providing us with a complexity range (e.g., 4–9). Within this range, we randomly sampled an explanation from each half-point (e.g., 4, 4.5, 5, 5.5, 6, 6.5, 7, 7.5, 8, 8.5, 9), providing us with eight to thirteen randomly sampled explanations for each version of each scenario. Participants were then randomly assigned to one version of each scenario and were randomly assigned to one corresponding explanation to evaluate, meaning approximately 25 participants viewed each explanation.

After reading each scenario and its accompanying explanation, participants were asked: How satisfied are you with the MTurker’s explanation? Questions were counterbalanced and assessed on a 1–9 Likert scale (first question: 1 = Extremely Dissatisfied, 9 = Extremely Satisfied; second question: 1 = Extremely Simple, 9 = Extremely Complex).

### 6.2. Results

Linear mixed modeling was used to analyze the results. Scenario complexity (simple, complex) and explanation complexity rating were included as fixed factors, along with the interaction between them. Participants and scenario complexity were entered as random factors.

The main effect of scenario complexity was statistically significant, *χ*^*2*^(1) = 18.35, *p* < .01. On average, participants were more satisfied with explanations for simple scenarios (*M* = 5.67, *SD* = 2.10) than they were for complex scenarios (*M* = 5.34, *SD* = 2.24). However, the interaction between scenario complexity and explanation complexity rating was also significant, *χ*^*2*^(1) = 11.52, *p* < .01. For every one-unit increase in explanation complexity rating, explanatory satisfaction for complex scenarios increased by 0.52-unit, while satisfaction for simple scenarios increased by 0.24-unit.

### 6.3. Discussion

Overall, participants tend to be more satisfied with an explanation as it increases in complexity [14]. The findings of the current study appear to align with the findings from Study 1 of Zemla et al. (2017) [14]. In that first study, Zemla and colleagues mined events and explanations from popular sites such as Reddit, and they asked participants to evaluate these explanations. The authors found a strong positive correlation between explanatory complexity and explanatory quality, even when controlling for the number of effects in the event to be explained. Thus, the current study seems to support a preference for explanatory complexity.

However, this relationship is stronger for complex scenarios than for simple scenarios, which the Zemla (et al., 2017) [14] account would not predict. This provides modest support for the complexity-matching hypothesis. The data pattern suggests two additive forces working in conjunction: a general preference for more complexity (as explanatory complexity increases, satisfaction increases for both simple and complex scenarios), along with a preference for complexity matching (satisfaction increases at a higher rate for complex scenarios).

The scenarios used here are more similar to the realistic stimuli used by Zemla et al. (2017) [14] rather than the simpler laboratory stimuli of Lombrozo (2007) [5]. While our scenarios did involve made-up events (e.g. a fictional baseball team), these are topics for which participants’ existing semantic memory would be relevant in evaluating the quality of the explanations (as opposed to the blank predicate structure of diagnosing alien diseases, cf. [5]). However, even under such conditions for which individuals may have a general bias towards complex explanations, it seems that this preference is still moderated by the complexity of the event at hand.

Both the current study and the work of Zemla et al. (2017) [14] relied on naturalistic, user-generated explanations as their main stimuli. While this has many advantages, especially in terms of providing a sense of external validity, it does carry disadvantages. Specifically, a lack of experimental control over the stimuli makes it hard to account for the variety of ways that the explanations can vary beyond complexity, or to ensure that the entire range of complexity is adequately sampled. For example, a post-hoc analysis of the MTurkers’ generated explanations showed that while the explanations varied on perceived complexity, they did not vary in parsimony–how we had operationalized complexity in the previous studies. Across the sampled explanations for each scenario, MTurkers tended to be similarly parsimonious, providing 1–2 causes for the event in question regardless of the complexity of the event that elicited those explanations. However, for explanations the raters perceived as being more complex, the MTurkers elaborated on their reasons, providing more in-depth descriptions, thus increasing *perceived* complexity despite the lack of variation in parsimony. Given that this differs from our previous operationalization of complexity, to ensure the robustness of the findings it seemed prudent to run an additional study using a different manipulation of complexity.

## 7. Study #4

The previous three studies all manipulated complexity in the same way: number of details (parsimony). However, as for all multifaceted constructs, any given operationalization of complexity will vary on multiple dimensions, leading to the possibility of confounds. For example, one could argue that differences in length underlie the results from previous studies, as longer scenarios may produce a demand effect, indirectly prompting people to expect and generate longer explanations. Alternatively, as more details are provided in a scenario, participants may believe that explanations would need to be longer in order to account for the extra information. Thus, to address these arguments and provide robust evidence for the complexity-matching hypothesis, Study 4 uses a fundamentally different operationalization of complexity—uniformity (the consistency of relationships among scenario details).

In the following study, we hold the number of scenario details constant (thus holding length constant), and manipulate uniformity by providing either valence consistent (details were either all positive or all negative) or inconsistent (a mix of positive and negative details) information. Previous research has shown that people often have trouble with reasoning through events featuring both positive and negative elements [52]. Given this, valence-inconsistent events should be perceived as more complex than valence-consistent events. As a result, the complexity-matching hypothesis would then predict that valence-inconsistent scenarios should merit more complex explanations than valence-consistent scenarios.

### 7.1. Method

#### 7.1.1. Participants

A sample of MTurk participants (n = 253) was used, comprised of 45% males, 55% females, with a mean age of 33.3 years. Sample size was based on the same calculations as for Studies 1 and 2. Once again, we excluded participants who had completed any of the previous three studies.

#### 7.1.2. Materials

The four domains from Study 1 were used (store, baseball team, employee, university). Each scenario had two details describing an event of interest, with the same two details appearing in all versions of the scenario. However, we manipulated each detail to be either positive (+) or negative (-) in valence. For example, the following detail regarding the university was (+):

“It was recently christened a top-twenty university by Canadian News & World Report, the first time the school had ever received such an honor.”

The (-) version of this detail then read:

“It was recently dropped from the list of top-twenty universities by Canadian News & World Report, the first time the school had ever been absent from the list.”

Because there were two details for each scenario, this resulted in a total of four possible versions of the scenario (see S2 Appendix). Two versions were valence consistent (++, --) and thus uniform, while two were valence inconsistent (+-, -+), and thus non-uniform. Participants read through all four scenarios, but were randomly assigned to only one of the four versions of each scenario.

As in Study 1, for each scenario, participants answered the question: “How complex do you think a satisfying explanation to this event will be?” (cf. [14]). Explanation complexity was assessed on a 1–9 Likert scale, anchored by 1 = Extremely Simple, 9 = Extremely Complex. As a manipulation check, participants were also asked to evaluate the complexity of the scenario that they had read, assessed on the same 1–9 Likert scale.

### 7.2. Results

As in the previous studies, linear mixed effects regression was used to analyze the dependent measures. The four possible versions of scenario valence were condensed into two levels (consistent, inconsistent), and this was entered as a fixed factor, along with scenario (store, baseball team, employee, university). The interaction between scenario valence and scenario was included as a third fixed factor. Participants and scenario valence were entered as random factors.

The manipulation check revealed that, as expected, participants found the inconsistent scenarios (*M* = 5.80, *SD* = 1.86) to be more complex than the consistent scenarios (*M* = 5.23, *SD* = 2.06), *χ*^*2*^(1) = 29.17, *p* < .001.

For evaluations of the necessary complexity of an explanation, analysis revealed a main effect of scenario valence, *χ*^*2*^(1) = 29.05, *p* < .001. Participants thought that inconsistent scenarios (*M* = 5.67, *SD* = 1.78) required more complex explanations than did consistent scenarios (*M* = 5.15, *SD* = 2.03). There was also a main effect of scenario, *χ*^*2*^(3) = 45.50, *p* < .001 (See Table 7), but no interaction between scenario valence and scenario, *χ*^*2*^(3) = 5.68, *p* = ns. Thus, in alignment with the complexity-matching hypothesis, participants predicted that valence-inconsistent scenarios would have more complex explanations than valence-consistent scenarios.

**Table 7:** Complexity of a satisfying explanation.

	Complex	Simple
MacGrady	6.04 (1.56)	5.27 (1.95)
Baseball	5.69 (1.64)	4.88 (2.02)
Employee	5.00 (1.92)	4.74 (2.06)
Friedman	5.95 (1.82)	5.71 (1.96)

Statistics represent mean(standard deviation).

### 7.3. Discussion

Study 4 generalized the findings across a novel operationalization of complexity (uniformity), with the finding remaining the same: people still exhibited a preference for complexity matching. In addition, because we held number of details constant in our scenarios, we were able to rule out many other factors besides complexity that may have contributed to the effect in previous studies.

Overall, across two different manipulations of complexity (number of details, valence consistency) and three different dependent measures (prediction, generation, evaluation), the results remain consistent with the complexity-matching hypothesis.

## 8. General discussion

For centuries, scholars have been trying to understand what makes for a satisfying explanation. Many factors have been found to contribute to the perceived quality of an explanation, from an explanation’s teleological properties [53–54], to less normatively defensible factors, such as the inclusion of neuroscience [55] and math [56], or appeals to particular scripts and norms [57]. More specifically, we add to the work on explanatory virtues, as first outlined by Thagard (1978) [3] and later carried on by other researchers in the domains of coherence [9, 58], breadth [10, 59], and simplicity [10, 5, 16].

In the current work, we focus on the role of simplicity. While many researchers have suggested that simplicity is an explanatory virtue, others have found evidence for the desirability of complexity in explanations. The current work attempts to resolve this apparent discrepancy by showing that both sides are partially correct. We demonstrate that individuals tend to prefer for events and explanations to match in terms of complexity–people favor simpler explanations for simple events, and more complex explanations for complex events.

The current work also adds to the emerging literature on matching in causal relationships [37–40]. Much of this previous work has examined pure cause-and-effect relationships. In the current work, we move the matching principle beyond the lens of cause-and-effect, and into explanations. While explanations and causal relationships are highly related, there are many factors unrelated to perceptions of causal relationships that contribute to the satisfactoriness of an explanation, such as the inclusion of reductive factors [60], the teleological structure [53–54], and even length [29]. Thus, to find that complexity matching also contributes to explanatory satisfaction is noteworthy.

Additionally, the past literature on matching has almost exclusively examined physical dimensions, such as magnitude and appearance. The fact that matching also occurs along abstract dimensions such as complexity is novel, and future research may find it worthwhile to explore the extent to which other abstract dimensions exhibit matching effects, in both explanatory and causal reasoning.

### Future directions

#### Defining simplicity

In the current work, we define simplicity in terms of two components: parsimony and uniformity. While the set of studies presented here examines these two factors in-depth, there are doubtless many other components comprising this (ironically complex) construct. Beyond the quantity and consistency of elements, one interesting direction for future examination comes from Study 2. While participants wrote explanations that were rated as more complex for complex scenarios as opposed to simple ones, there was no difference in number of causes described between the two conditions (as would be predicted by parsimony). However, post-hoc analysis of participants’ explanations shows that for complex scenarios, participants often went into greater depth in explaining the causes they listed, providing more nuance and elaboration of their thoughts. The fact that explanations for complex scenarios featured higher word counts and Flesch-Kincaid scores would seem to support this intuition, and this is an avenue worth examining further in future work. We encourage others to explore conceptualizations of simplicity further, and a standardized taxonomy of what this construct entails should provide exciting new avenues of research.

#### Explanatory goals

In the current work, we implicitly assume that people’s goal in explanation is to gain a sense of understanding [61–62]. However, beyond understanding, there are a variety of other goals that individuals may adopt with regard to explanation, such as prediction and control [63–65], the desire to “fix” something broken [66], discovery of underlying causal structure [67–69], data interpretation [70], or simply to obtain a sense of satisfaction [65]. It is worth asking how people’s preferences for simplicity or complexity in explanation will change as their goals shift. For example, if prediction and control is the objective, then individuals may exhibit a stronger preference for complexity, as figuring out the nuances of a situation should allow for greater projection of potential outcomes. Thus, future researchers are encouraged to think about the goals individuals have when seeking out explanation, and how these aims may affect their explanatory preferences.

#### Characteristics of the event

We examined a key attribute of the precipitating event–complexity–which affected people’s explanatory preferences. However, we do not argue that complexity is the only characteristic of an event that matters. For example, previous research has demonstrated that individuals are more likely to think about and elaborate on events that they consider self-relevant or important [71]. It is plausible that the importance or relevance of the event being explained would similarly lead to differences in preferences regarding explanatory complexity, with more important events meriting more complex explanations. Indeed, there are potentially many other characteristics of a precipitating event that could influence people’s explanatory preferences, and future research would do well to explore this area further.

#### Identifying mechanism

While the current work has provided robust evidence for the phenomenon of complexity matching, up to this point we have not been able to address the issue of mechanism–*why* do people complexity-match to begin with? One possible answer can be found in work on probability/likelihood tradeoffs [72–73]. Johnson and colleagues (2017) [72] argue that explanations tend to vary in their probability, with simpler explanations being more probable than complex ones. However, complex explanations are often better fits for the event at hand, as they are better able to account for the totality of data presented (i.e. the Bayesian likelihood is higher). Thus, attributing a university’s success to the confluence of hiring new faculty, adding new majors, and receiving large endowments is a more powerful explanation than any one of these factors alone would be, but having the three factors all occur is much less probable than having any single factor take place. As a result, individuals must trade off the probability and likelihood of an explanation in order to obtain an optimal balance for any given event.

Extending Johnson’s model to the present findings, it could be, as Lombrozo (2007) [5] and others have argued [8, 10, 15–16], that people have a natural preference for simplicity as simpler explanations have higher probability. However, while people are always looking for the simplest explanation possible, complex data may not be adequately fit by any simple explanation, leaving only complex explanations as viable. Managing the tradeoff between probability and likelihood leads people to adopt more complex explanations as the complexity of the event increases.

We conducted several preliminary studies to test this proposed tradeoff. An initial pre-test showed that people do indeed view simple explanations as having higher probability (but lower likelihood) in comparison to complex explanations. However, subsequent studies run to explicitly test this tradeoff have consistently revealed uninterpretable results. As such, while we have robustly demonstrated a phenomenon (complexity matching) that speaks to what makes for a satisfying explanation, we have ironically been unable to establish a satisfying explanation for this phenomenon. This should serve as the impetus for future research.

#### Practical implications

Given the fact that explanations are a pervasive part of everyday life, these findings also have implications across a wide variety of fields, such as medicine, law, and marketing. For example, the American Medical Association notes that one major reason why many patients fail to take their medication is a lack of understanding—“patients may not understand the need for the medicine, the nature of the side effects, or the time it will take to achieve results” [74]. Medical practitioners may thus have better success in reaching their patients if they were to better tailor explanations to patients’ perceptions of complexity. Similarly, in law, attorneys may do well to adapt the complexities of their explanations and arguments to the jury’s desired level of complexity. In marketing, many public relations crises involve complex events and outcomes which might make simple explanations seem inadequate, or worse, insulting. Public relations officers and brand managers would thus do well to think about the complexity of the crisis, and to fashion their explanations accordingly.

In the end, our work sheds light on the question of what constitutes a satisfying explanation by providing complexity matching as a factor worthy of further study. However, there is still much more work to be done to explain explanations. Simply put, the answer to this seemingly simple question appears to be quite complex.

## Supporting information

S1 Appendix (DOCX)

S2 Appendix (DOCX)
