# Supplementary material for: Explanatory preferences for complexity matching
Source: PLoS One. 2020 Apr 21;15(4):e0230929. doi: 10.1371/journal.pone.0230929 (PMC7173929; doi:10.1371/journal.pone.0230929)
Supplement: S1 Appendix — (DOCX) [file pone.0230929.s001.docx]

Appendix A

**“Store”**

**Complex Event**

MacGrady Co. is a seller of outdoor gear. This year has been a very successful one for the company. Consumer

interest rose, with an all-time high of 80% of its target customer base acknowledging that they would consider

shopping at MacGrady. Word-of-mouth for the brand also increased as well, with 30% more mentions on social media sites such as Facebook and Twitter. Additionally, consumer traffic in MacGrady’s stores also rose by 25%, the largest increase for the company in the past ten years.

**Simple Version #1**

MacGrady Co. is a seller of outdoor gear. This year has been a very successful one for the company. Consumer interest rose, with an all-time high of 80% of its target customer base acknowledging that they would consider shopping at MacGrady.

**Simple Version #2**

MacGrady Co. is a seller of outdoor gear. This year has been a very successful one for the company. Word-of-mouth for the brand increased, with 30% more mentions on social media sites such as Facebook and Twitter.

**Simple Version #3**

MacGrady Co. is a seller of outdoor gear. This year has been a very successful one for the company. Consumer traffic in MacGrady’s stores rose by 25%, the largest increase for the company in the past ten years.

**“Company”**

**Complex Version**

“Friedman University has been having a great year. It was recently christened a top-twenty university by Canadian News & World Report, the first time the school had ever received such an honor. Additionally, upon graduation, 90% of Friedman’s senior class this year will either be employed or attending graduate school. On top of this, the entering freshman class looks to be very strong, with an average high school GPA of 3.98 (out of 4.00).”

**Simple Version #1**

“Friedman University has been having a great year. It was recently christened a top-twenty

university by Canadian News & World Report, the first time the school had ever received such

an honor.”

**Simple Version #2**

“Friedman University has been having a great year. Upon graduation, 90% of Friedman’s senior class this year will either be employed or attending graduate school.”

**Simple Version #3**

“Friedman University has been having a great year. The entering freshman class looks to be very strong, with an average high school GPA of 3.98 (out of 4.00).”

**“Baseball Team”**

**Complex Event**

The baseball program at Northeastern Oregon University has recently experienced some very difficult times, despite owning three national championships in its storied history. The team has only managed to win more than half of its games twice in the past seven years. Additionally, the team has only had three players drafted by the MLB in that time frame, after having had fifteen players drafted in the seven years prior. Furthermore, during the past seven years, at least a dozen players have chosen to either leave the program or transfer to another school, which is unprecedented for a powerhouse program like Northeastern Oregon.

**Simple Version #1**

The baseball program at Northeastern Oregon University has recently experienced some very difficult times, despite owning three national championships in its storied history. The team has only managed to win more than half of its games twice in the past seven years.

**Simple Version #2**

The baseball program at Northeastern Oregon University has recently experienced some very difficult times, despite owning three national championships in its storied history. The team has not had any players drafted by the MLB in the past seven years, after having had fifteen players drafted in the seven years prior.

**Simple Version #3**

The baseball program at Northeastern Oregon University has recently experienced some very difficult times, despite owning three national championships in its storied history. During the past seven years, at least a dozen players have chosen to either leave the program or transfer to another school, which is unprecedented for a powerhouse program like Northeastern Oregon.

**“Employee”**

**Complex Event**

Gus seems to be in some sort of funk. At work, his sales numbers have gone down, as he has been aloof, and even cold, towards clients. To make matters worse, he has become extremely difficult to be around, often sulking and grumbling in front of his colleagues. Furthermore, he has refused to take part in any of the office’s usual social functions, such as its potlucks or bowling nights, events he used to be eager to attend and even help plan.

**Simple Version #1**

Gus seems to be in some sort of funk. At work, his sales numbers have gone down, as he has been aloof, and even cold, towards clients.

**Simple Version #2**

Gus seems to be in some sort of funk. At work, he has become extremely difficult to be around, often blowing up in front of his colleagues.

**Simple Version #3**

Gus seems to be in some sort of funk. At work, he has refused to take part in any of the office’s usual social functions, such as its potlucks or bowling nights, events he used to be eager to attend and even help plan.
