# Supplementary material for: Explanatory preferences for complexity matching
Source: PLoS One. 2020 Apr 21;15(4):e0230929. doi: 10.1371/journal.pone.0230929 (PMC7173929; doi:10.1371/journal.pone.0230929)
Supplement: S2 Appendix — (DOCX) [file pone.0230929.s002.docx]

Appendix B

**“Store”**

(+ +)

MacGrady Co. is a seller of outdoor gear. This year has been a very interesting one for the company. Consumer interest rose, with an all-time high of 70% of its target customer base acknowledging that they would consider shopping at MacGrady. Additionally, consumer traffic in MacGrady’s stores rose by 25%, the largest increase for the company in the past ten years.

(- -)

MacGrady Co. is a seller of outdoor gear. This year has been a very interesting one for the company. Consumer interest declined, with an all-time low of 30% of its target customer base acknowledging that they would consider shopping at MacGrady. Additionally, consumer traffic in MacGrady’s stores dropped by 25%, the largest decrease for the company in the past ten years.

(+ -)

MacGrady Co. is a seller of outdoor gear. This year has been a very interesting one for the company. Consumer interest rose, with an all-time high of 70% of its target customer base acknowledging that they would consider shopping at MacGrady. However, consumer traffic in MacGrady’s stores dropped by 25%, the largest decrease for the company in the past ten years.

(- +)

MacGrady Co. is a seller of outdoor gear. This year has been a very interesting one for the company. Consumer interest declined, with an all-time low of 30% of its target customer base acknowledging that they would consider shopping at MacGrady. However, consumer traffic in MacGrady’s stores rose by 25%, the largest increase for the company in the past ten years.

**“University”**

**(+ +)**

“Friedman University has been having an interesting year. It was recently christened a top-twenty university by Canadian News & World Report, the first time the school had ever received such an honor. Additionally, upon graduation, 90% of Friedman’s senior class this year will either be employed or attending graduate school, the highest such rate in Friedman’s history.”

**(- -)**

“Friedman University has been having an interesting year. It was recently dropped from the list of top-twenty universities by Canadian News & World Report, the first time the school had ever been absent from the list. Additionally, upon graduation, only 40% of Friedman’s senior class this year will either be employed or attending graduate school, the lowest such rate in Friedman’s history.”

**(+ -)**

Friedman University has been having an interesting year. It was recently christened a top-twenty university by Canadian News & World Report, the first time the school had ever received such an honor. However, upon graduation, only 40% of Friedman’s senior class this year will either be employed or attending graduate school, the lowest such rate in Friedman’s history.

**(- +)**

Friedman University has been having an interesting year. It was recently dropped from the list of top-twenty universities by Canadian News & World Report, the first time the school had ever been absent from the list. However, upon graduation, 90% of Friedman’s senior class this year will either be employed or attending graduate school, the highest such rate in Friedman’s history.

**“Baseball Team”**

(+ +)

The baseball program at Northeastern Oregon University has recently experienced some very interesting years. The team has had fifteen players drafted by the MLB in the past seven years, after having had no players drafted in the seven years prior. Furthermore, booster support has increased by 30%, as more donors have been willing to financially support the team.

(- -)

The baseball program at Northeastern Oregon University has recently experienced some very interesting years. The team has not had any players drafted by the MLB in the past seven years, after having had fifteen players drafted in the seven years prior. Furthermore, booster support has decreased by 30%, as fewer donors have been willing to financially support the team.

(+ -)

The baseball program at Northeastern Oregon University has recently experienced some very interesting years. The team has had fifteen players drafted by the MLB in the past seven years, after having had no players drafted in the seven years prior. Unfortunately though, booster support has decreased by 30%, as fewer donors have been willing to financially support the team.

(- +)

The baseball program at Northeastern Oregon University has recently experienced some very interesting years. The team has not had any players drafted by the MLB in the past seven years, after having had fifteen players drafted in the seven years prior. Fortunately though, booster support has increased by 30%, as more donors have been willing to financially support the team.

**“Employee”**

(+ +)

Gus’s behavior has been very interesting lately. At work, his sales numbers have gone up, as he has been friendlier and more personable towards clients. Furthermore, he has been excited to take part in many of the office’s extracurricular activities, such as community service events and the annual basketball league, things he used to hate to do.

(- -)

Gus’s behavior has been very interesting lately. At work, his sales numbers have gone down, as he has been aloof, and even cold, towards clients. Furthermore, he has refused to take part in any of the office’s extracurricular activities, such as community service events or the annual basketball league, things he used to love to do.

(+ -)

Gus’s behavior has been very interesting lately. At work, his sales numbers have gone up, as he has been friendlier and more personable towards clients. However, he has refused to take part in any of the office’s extracurricular activities, such as community service events or the annual basketball league, things he used to love to do.

(- +)

Gus’s behavior has been very interesting lately. At work, his sales numbers have gone down, as he has been aloof, and even cold, towards clients. However, he has been excited to take part in many of the office’s extracurricular activities, such as community service events and the annual basketball league, things he used to hate to do.
